# Cell cycle status of male and female gametes during Arabidopsis reproduction

**DOI:** 10.1093/plphys/kiad512

**Published:** 2023-09-27

**Authors:** Yoav Voichek, Bohdana Hurieva, Caroline Michaud, Anna Schmücker, Zaida Vergara, Bénédicte Desvoyes, Crisanto Gutierrez, Viktoria Nizhynska, Benjamin Jaegle, Michael Borg, Frédéric Berger, Magnus Nordborg, Mathieu Ingouff

**Affiliations:** Gregor Mendel Institute, Austrian Academy of Sciences, Vienna Biocenter (VBC), Vienna, Austria; Gregor Mendel Institute, Austrian Academy of Sciences, Vienna Biocenter (VBC), Vienna, Austria; DIADE, IRD, CIRAD, University Montpellier, Montpellier, France; Gregor Mendel Institute, Austrian Academy of Sciences, Vienna Biocenter (VBC), Vienna, Austria; Centro de Biología Molecular Severo Ochoa, CSIC-UAM, Madrid, Spain; Centro de Biología Molecular Severo Ochoa, CSIC-UAM, Madrid, Spain; Centro de Biología Molecular Severo Ochoa, CSIC-UAM, Madrid, Spain; Gregor Mendel Institute, Austrian Academy of Sciences, Vienna Biocenter (VBC), Vienna, Austria; Gregor Mendel Institute, Austrian Academy of Sciences, Vienna Biocenter (VBC), Vienna, Austria; Department of Algal Development and Evolution, Max Planck Institute for Biology, Tübingen, Germany; Gregor Mendel Institute, Austrian Academy of Sciences, Vienna Biocenter (VBC), Vienna, Austria; Gregor Mendel Institute, Austrian Academy of Sciences, Vienna Biocenter (VBC), Vienna, Austria; DIADE, IRD, CIRAD, University Montpellier, Montpellier, France

## Abstract

Fertilization in Arabidopsis (*Arabidopsis thaliana*) is a highly coordinated process that begins with a pollen tube delivering the 2 sperm cells into the embryo sac. Each sperm cell can then fertilize either the egg or the central cell to initiate embryo or endosperm development, respectively. The success of this double fertilization process requires a tight cell cycle synchrony between the male and female gametes to allow karyogamy (nuclei fusion). However, the cell cycle status of the male and female gametes during fertilization remains elusive as DNA quantification and DNA replication assays have given conflicting results. Here, to reconcile these results, we quantified the DNA replication state by DNA sequencing and performed microscopic analyses of fluorescent markers covering all phases of the cell cycle. We show that male and female Arabidopsis gametes are both arrested prior to DNA replication at maturity and initiate their DNA replication only during fertilization.

## Teaser

Mature male and female Arabidopsis gametes arrest their cell cycle before DNA replication, which only starts during fertilization.

## Introduction

Sexual reproduction in flowering plants involves key developmental phases that first generate genetically distinct haploid spores during meiosis (Kawashima et al. 2014). After several rounds of division, the male and female spores differentiate into 2 male gametes (sperm cells) within a pollen grain and 2 female gametes (egg cell, central cell) inside an embryo sac. During double fertilization, 1 sperm cell fuses with the egg cell to give rise to the embryo, while the second fuses with the diploid central cell to form the endosperm. This developmental mechanism, which is typical of flowering plants, requires a tight synchronization of the cell cycle between the male and the female gametes to ensure successful fusion.

Quantification of DNA content using 4,6-diamidino-2-phenylindole (DAPI) staining has suggested that sperm cells are arrested during DNA replication in Arabidopsis (*Arabidopsis thaliana*) pollen grains (Friedman 1999; Rotman et al. 2005; Kim et al. 2008) and reach the G2 phase only at fertilization (Friedman 1999). However, recent experiments using 5-ethynyl-2′-deoxyuridine (EdU) staining to detect active DNA replication in pollen grains and in growing pollen tubes of Arabidopsis failed to detect any labeling consistent with a cell-cycle arrest prior to DNA replication in sperm cells (Liu et al. 2021). In addition, the phase of cell-cycle arrest of female gametes (egg cell and central cell) has remained largely unexplored. Measurements of nuclear DNA content suggested that both female gametes are arrested prior to DNA replication (Mogensen et al. 1995; Mogensen and Holm 1995; Tian et al. 2005; Sukawa and Okamoto 2018). Overall, the cell cycle state of the male and female gametes during fertilization is still unclear as the current methods employed, such as DNA quantification and DNA replication assays, have given inconsistent results (Friedman 1999; Rotman et al. 2005; Kim et al. 2008; Liu et al. 2021).

Here, to assess the cell cycle status of the male and female gametes during the fertilization, we combined 2 strategies as follows: DNA sequencing (DNA-Seq) and microscopic examination of fluorescent cell-cycle markers. Our findings reveal that the cell cycle of both male and female Arabidopsis gametes is blocked prior to DNA replication, and that DNA replication is triggered only during fertilization.

## Results

### Mature sperm cells are arrested in a pre-replication phase until fertilization

**Figure 1.**
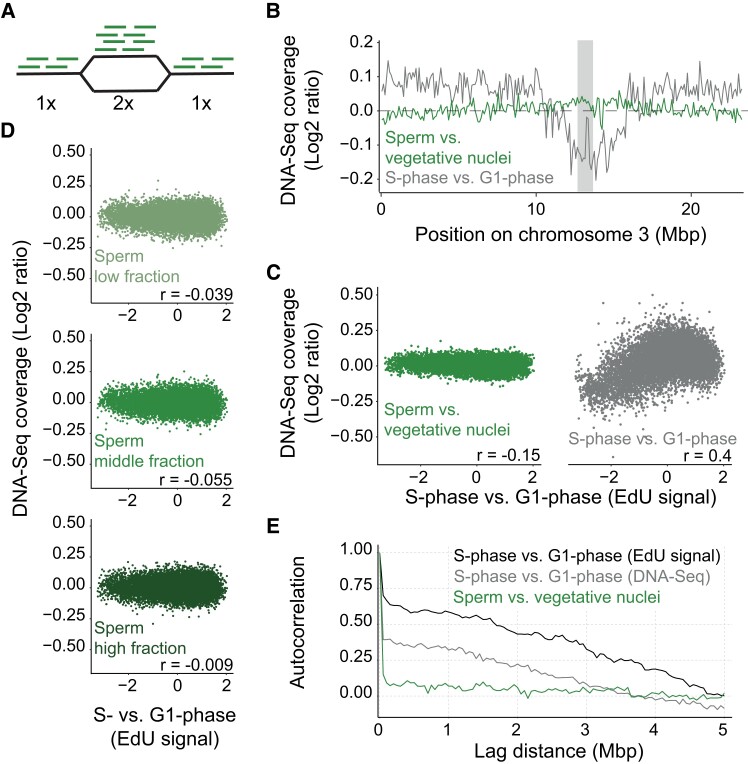
Quantification of DNA replication in sperm nuclei by DNA-Seq. **A**) Replicated regions yield double the coverage in DNA-Seq relative to non-replicated regions. Black line, DNA; green line, sequence reads; 1x, non-replicated; 2x, replicated regions. **B**) DNA-Seq coverage along chromosome 3 in sperm vs. vegetative nuclei (green) or of cells enriched for S vs. G1 phase (gray). Coverage is averaged over 100 kbp bins and ratios are log2 transformed; shaded gray denotes the centromere position. **C**) Correlation between replication timing, as measured by EdU enrichment from early S-phase cells (Concia et al. 2018), to DNA-Seq coverage in sperm vs. vegetative nuclei (left) or of cells in S phase vs. G1 phase (right). Each dot is an average of 10 kbp bin in the genome, and Pearson's correlation coefficient values are shown. **D**) Correlation of DNA-Seq coverage to replication timing as in **C**), for the 3 fractions of sperm nuclei sorted based on PI staining intensity, from lowest (top) to highest (bottom). **E**) Autocorrelation of the genomic signal to itself for different distances, for early S vs. G1 phase enriched with EdU (Concia et al. 2018) (black), S vs. G1 phase quantified with DNA-Seq (gray) and sperm vs. vegetative nuclei quantified with DNA-Seq (green). The genome was averaged over 10 kbp bins. All next-generation sequencing data used in this figure are listed in Supplemental Table S1.

As a first step, we adapted a DNA-Seq approach (Voichek et al. 2016) that can detect even subtle DNA replication signals (Fig. 1A). To demonstrate the ability of the DNA-Seq to detect DNA replication in Arabidopsis, we performed a control experiment where suspension cells were enriched for G1 or S phase (Supplemental Fig. S1). Genome-wide coverage in S- vs. G1-phase cells showed an increase along chromosome arms, as expected from early replicating regions (Fig. 1B, gray curve). Next, we applied our DNA-Seq approach to sperm and vegetative nuclei isolated from mature pollen grains using fluorescence-activated cell sorting (FACS) (Kawashima et al. 2014). We first compared the genome-wide coverage of purified sperm nuclei with that of vegetative nuclei or root nuclei. In contrast to our control experiments, the coverage was uniform along the chromosomes (Fig. 1B, green curve; Supplemental Fig. S2). Although the genome-wide coverage pattern showed no evidence of DNA replication, a subpopulation of sperm cells may still be replicating their DNA. This would be evident as a weaker signal of DNA replication when compared with previously published replication data from Arabidopsis (Concia et al. 2018). However, we observed no correlation with published profiles of DNA replication timing, as opposed to in the control experiment (Fig. 1C). We also assessed this possibility by sorting 3 subpopulations of sperm cells based on increasing propidium iodide (PI) staining, which might reflect increased DNA replication (Supplemental Fig. S3A to C). Regardless of the PI fluorescence, sperm nuclei never showed a signal of DNA replication in our DNA-Seq measurements (Fig. 1D). Lastly, we also ruled out the possibility that profiles of DNA replication timing in sperm cells might differ from that published for other cell types. Genomic DNA-replication profiles are assumed to be continuous, since close-by regions should replicate at similar times. Therefore, autocorrelation of the coverage between near-by positions should correlate if DNA is replicating, irrespective of the replication order. In contrast to the synchronized cells, no autocorrelation was observed for sperm cells (Fig. 1E; Supplemental Fig. S3D).

Thus, our genome-wide analysis could not detect any DNA replication in sperm, contrary to published results (Friedman 1999; Rotman et al. 2005; Kim et al. 2008). Our analyses suggest that DAPI-based measurement of DNA content is not a reliable method to assess cell cycle phase in sperm nuclei, probably due to their peculiar chromatin structure (Liu et al. 2021). However, direct measurements using DNA sequencing could not determine directly if sperm cells in mature pollen were arrested in G1 or G2 phase.

**Figure 2.**
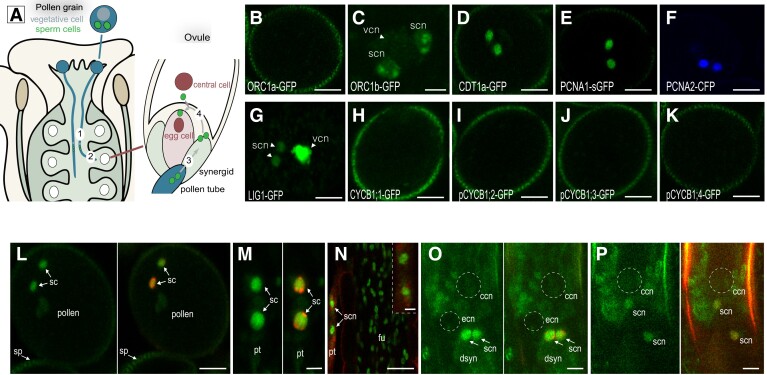
Mature sperm cells are arrested in a pre-replication phase until fertilization. **A**) Schematic representation of key steps leading to double fertilization. Migration of sperm cells (green) in the growing pollen tube (blue) through the female sporophyte (step 1), along the funiculus toward the ovules (step 2), release of the sperm cells into the embryo sac (step 3), and migration toward the female gametes (egg cell and central cell, red, step 4). **B to K**) Expression pattern of fluorescent cell-cycle phase markers in mature pollen grains. No expression of the G1-phase marker ORC1a-GFP **B**). Punctate fluorescent foci in sperm nuclei and weak uniform fluorescence in the vegetative cell expressing the G1 phase marker ORC1b-GFP **C**). The pre-replication marker CDT1a-GFP is expressed in the sperm cells **D**). The PCNA1-sGFP **E**), PCNA2-CFP **F**), and LIG1-GFP **G**) markers form nuclear foci during DNA replication and a whole nuclear pattern during the G1 and G2 phases (Yokoyama et al. 2016). A diffuse fluorescence accumulates for the 3 markers in the sperm nuclei. No fluorescence is detected for all the 4 G2/M markers (CYCB1;1-GFP **H**); pCYCB1;2-GFP **I**); pCYCB1;3-GFP **J**); pCYCB1;4-GFP **K**). Further details on the dynamics of these cell-cycle markers are provided in Supplemental Fig. S4. Number of observations *n* > 25 for each marker. **L to P**) Dynamics of PCNA1-sGFP (green) subnuclear localization **N**) or in combination with a histone 1 heterochromatin fluorescent marker (H1-1-RFP, red) **L to P**) during all the key steps of sperm cell migration described in **A**). The pollen tube is visualized by autofluorescence (red) **N**). **L**) Diffuse PCNA1-GFP fluorescence in sperm cell nuclei upon pollination, during pollen tube growth through the style (**M**, step 1), along the funiculus (**N**, step 2), and after discharge in 1 synergid of the embryo sac (**O**, step 3). A weaker uniform fluorescence is detected upon fertilization (**P**, step 4). The signals around the central cell nucleus in panels **O**) and **P**) correspond to autofluorescence. Numbers of observations *n* = 20 **L**), *n* = 12 **M**), *n* = 2 **N**), *n* = 5 **O,P**). ccn, central cell nucleus; dsyn, degenerated synergid; ecn, egg cell nucleus; fu, funiculus; pt, pollen tube; sc, sperm cell; scn, sperm cell nucleus; sp, stigmatic papillae; vcn, vegetative cell nucleus. Scale bars, 10 *µ*m **B to L,N**), 5 *µ*m (**M**,inset in **N**,**O**,**P**).

Given this, we sought to determine when sperm cells do commence DNA replication. We selected Arabidopsis lines expressing fluorescent markers of key components of the G1, S, and G2/M phases fused with a gene encoding a fluorescent protein. Confocal analyses of root cells combining markers of G1 and S phases, of S and G2/M phases, and of the different S-phase markers revealed dynamics and subnuclear patterns that can trace distinct phases of the cell cycle (Supplemental Fig. S4). We therefore analyzed their pattern in sperm cells in mature pollen grains (Fig. 2A to K). We did not detect fluorescence for ORC1a-GFP, a component of the origin recognition complex (ORC), (Fig. 2B) in sperm cell nuclei whereas ORC1b-GFP (Fig. 2C), which is degraded at the G1/S transition in proliferating cells, showed a punctate pattern consistent with that of meristematic and early differentiating root tip cells (Vergara et al. 2023). Likewise, ORC2-GFP, which is also acting in G1, was detected in sperm nuclei (Supplemental Fig. S5A). The Chromatin licensing and DNA replication factor 1 (CDT1a), another component of the pre-replication complex (pre-RC), that accumulates in G1 and is degraded at the G1/S transition (Desvoyes et al. 2019), was detected in sperm cells (Fig. 2D). Proliferating cell nuclear antigen (PCNA) and DNA ligase 1 (LIG1) are essential DNA replication factors (Moldovan et al. 2007; De Sanchez et al. 2012). Both factors form dotted foci during early replication and speckled foci during late replication but distributed uniformly in the nucleus in G1 and G2 phases in Arabidopsis (Yokoyama et al. 2016) (Supplemental Fig. S4). This triphasic pattern represents highly resolute visual information for the progression of S phase. Only a uniform fluorescence signal could be observed in sperm cells from plants expressing either of the 2 tagged *PCNA* genes present in the Arabidopsis genome (Fig. 2, E and F) or LIG1-GFP (Fig. 2G) (Andreuzza et al. 2010), suggesting a cell-cycle arrest in the G1 phase prior to DNA replication. To expand on the above observations, we analyzed the dynamics of the 4 B1-type cyclins (CYCB1) present in the Arabidopsis genome, which mark the G2/M phase (Weimer et al. 2016). As reported previously, CYCB1;1 and CYCB1;2 reporter lines showed no expression in sperm cells (Brownfield et al. 2009; Zheng et al. 2011) (Fig. 2H to I). Reporter lines of CYCB1;3 and CYCB1;4 which both include the upstream region of the gene up to the coding sequence encoding the G2/M destruction box fused to the *GFP* gene (Ubeda-Tomás et al. 2009; Weimer et al. 2016) also showed no detectable fluorescence (Fig. 2, J and K). These analyses suggest that sperm chromatin is not replicated at anthesis but is arrested in late G1, in agreement with our DNA-Seq analysis. Interestingly, none of the markers tested were detected in the vegetative cell with exception of a strongly uniform nuclear fluorescence of LIG1-GFP (Fig. 2, C and G), and a faint signal of ORC1b-GFP. Our data are consistent with the vegetative cell having exited the cell cycle and being in a quiescent state (Berger and Twell 2011).

Next, we searched for PCNA1-sGFP replicating foci in sperm cell nuclei from the time of pollination until their fusion with the female gametes at karyogamy (Fig. 2A). To facilitate these observations, we crossed wild type pistils with pollen from plants expressing PCNA1-sGFP and fluorescent histone 1 (H1-1-RFP) heterochromatin markers (Rutowicz et al. 2019) (except for Fig. 2N where only PCNA1-sGFP marker was used). Sharp heterochromatin foci of H1-1-RFP were detectable during all phases of sperm cell migration (Fig. 2L, M, O, and P). In contrast, only a diffuse fluorescent pattern of PCNA1-sGFP was observed in sperm cell nuclei during pollination and pollen tube growth (Fig. 2L to M). Semi in vivo pollination of wild type pistils with pollen grains expressing PCNA1-sGFP or ORC1b-GFP and CDT1a-RFP further confirmed that sperm DNA does not replicate during pollen tube growth (Supplemental Fig. S6, A and B). PCNA1-sGFP foci were still not observed upon release and migration of the sperm cells within the embryo sac (Fig. 2, O and P). Although PCNA1-sGFP fluorescence becomes difficult to detect at plasmogamy (the plasma membrane fusion of gametes), we did not distinguish any foci in the sperm cells under our imaging conditions indicating that sperm cells are delivered in the G1 phase. In conclusion, our results suggest that sperm cells are arrested in the G1 phase until they enter the ovules where they initiate DNA replication.

### The mature egg cell and central cell are arrested in a pre-replicating phase and only start replicating at fertilization

**Figure 3.**
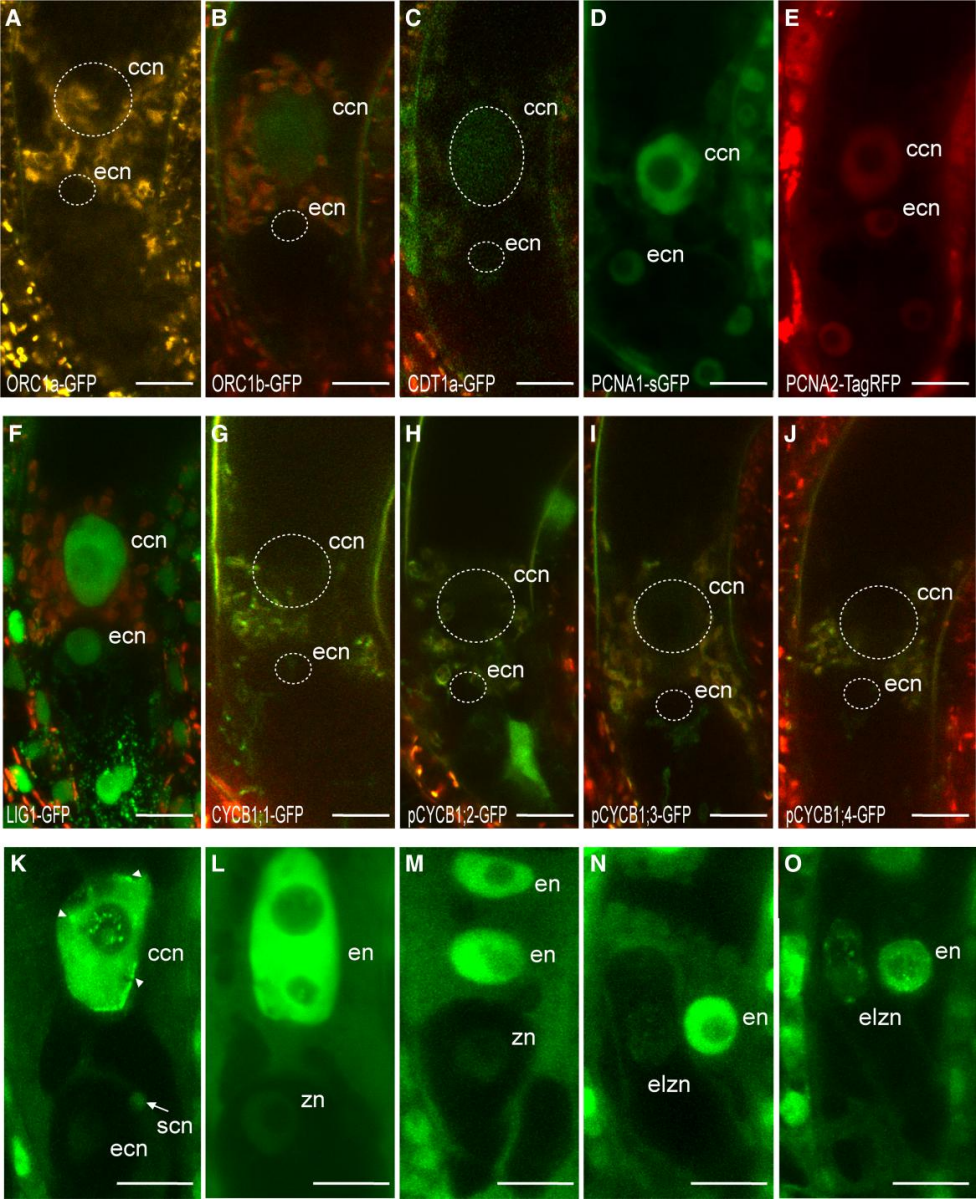
The mature egg cell and central cell are arrested in a different phase of the cell cycle before DNA replication and only start replicating at fertilization. **A to J**) Expression pattern of cell-cycle phase markers in the mature female gametes. The G1-phase marker ORC1a-GFP **A**) is not expressed in the female gametes. A weak fluorescence (green) of the G1 phase marker ORC1b-GFP **B**) and the pre-replication marker CDT1a-GFP (green) is detected specifically in the central cell nucleus **C**). In contrast to fluorescent nuclear foci formed during the S phase (Yokoyama et al. 2016) (Supplemental Fig. S1), a diffuse fluorescence of the 3 S-phase markers PCNA1-sGFP (green) **D**), PCNA2-TagRFP (red) **E**), and LIG1-GFP (green) **F**) markers is detected in both female gametes. No expression is detected for all the 4 G2/M markers CYCB1;1-GFP **G**); pCYCB1;2-GFP **H**); pCYCB1;3-GFP **I**); and pCYCB1;4-GFP **J**). The signals around the central cell nucleus and in the integuments correspond to autofluorescence (red) in panels **A to D**) and **F to J**). Further details on the dynamics of these cell-cycle markers can be found in Supplemental Fig. S1. Number of observations *n* > 25 for each marker. **K to O**) Dynamics of the PCNA1-sGFP subnuclear pattern in the developing zygote and endosperm. The egg cell and the central cell are fertilized by any of the 2 sperm cells to generate a zygote and an endosperm, respectively. **K**) The unfertilized egg cell nucleus retains a homogeneous nuclear fluorescence. The arrow indicates 1 sperm cell reaching the egg cell. Speckled foci of fluorescence (arrowheads) are detected in the central cell nucleus. **L**) The zygote and the forming endosperm with 2 nucleoli, both show a uniform nuclear fluorescence. **M**) The elongating zygote retains a uniform nuclear fluorescence through the 2-nucleate endosperm. **N,O**) A speckled nuclear pattern becomes detectable in the elongated zygote through the 4-nucleate and 8-nucleate endosperm. A complete view of panels **N**) and **O**) is shown in Supplemental Fig. S8. Number of observations *n* = 5 **K**), *n* = 6 **L**), *n* = 2 **M**), *n* = 5 **N,O**). ccn, central cell nucleus; ecn, egg cell nucleus; elzn, elongated zygote nucleus; en, endosperm nucleus; zn, zygote nucleus. Scale bars, 15 *µ*m **A to J**) and 10 *µ*m **K to O**).

Little is known about the cell-cycle arrest phase in the 2 types of female gametes: the egg cell and central cell. Gene expression analyses of cell-cycle-related genes in transcriptomes of purified Arabidopsis female gametes (Zhao et al. 2019; Susaki et al. 2021) showed no enrichment of phase-specific genes (Supplemental Table S2). Female gametes are limited in number and form deep within the pistil, and therefore, isolating enough for DNA-Seq is challenging. We therefore analyzed the expression pattern of our set of cell-cycle markers in the female gametes (Fig. 1A; Fig. 3). Of all the G1 markers, only a weak fluorescence could be detected for ORC1b-GFP and CDT1a-GFP in the central cell (Fig. 3A to C; Supplemental Fig. S5). All 3 S-phase markers (LIG1-GFP, PCNA1-sGFP, and PCNA2-TagRFP) accumulated nuclear fluorescence in both gametes without any detectable foci (Fig. 3D to F). In addition, G2/M markers were not detected in the female gametes (Fig. 3, G to J) (Ingouff et al. 2006; Johnston et al. 2008; Guo et al. 2016). To complement these observations, we analyzed the pattern of 2 cell cycle fluorescent sensors in the mature female gametes (Echevarría et al. 2021). The Plant Cell Cycle Indicator (PlaCCI) (Desvoyes et al. 2020) combines 3 genetic fusions—CDT1a-eCFP, CYCB1;1-YFP, and H3.1-mCherry, each of which indicate the G1, G2/M, and G1-to-G2/M phases, respectively. Similar to our previous observations (Fig. 3), we only observed a weak signal of CDT1a-eCFP in the central cell using the PlaCCI markers (Supplemental Fig. S7). The second fluorescent marker reports cells in S-G2-M (R Jones et al. 2017) and contains a VENUS fluorescent protein fused to a destruction box (DB) sequence from Arabidopsis *CYCB1;1* placed under the control of a histone H4 promoter. No fluorescence was detected in the mature embryo sac (Supplemental Fig. S7). We conclude that the central cell is arrested in a pre-replication phase. Because none of the markers we used were informative in the egg cell, we propose that it is in quiescent state (G0 phase). In contrast to other eukaryotes (Adikes et al. 2020; Pulianmackal et al. 2021), no specific G0 phase markers have been described so far in plants.

To track the release of the cell-cycle block in female gametes, we performed a time-course fertilization analysis of self-pollinated plants expressing PCNA1-sGFP using the precise developmental stages previously described (Faure et al. 2002). During plasmogamy which takes place about 1 h after release of the gametes in the embryo sac (Faure et al. 2002), speckled foci of PCNA1-sGFP were already evident in the elongated central cell nucleus but not in the egg cell nucleus (Fig. 3K). This PCNA1 pattern indicates late S phase that lasts approximately 1 to 1.5 h in somatic cells (Yokoyama et al. 2016). Early S-phase events were not detected in female gametes because the dotted pattern of PCNA1-GFP produces much less resolvable foci than speckles. Overall, our observations suggest that DNA replication in the central cell occurs upon sperm entry as previously reported (Aw et al. 2010). At a later stage, the fertilized central cell nucleus contained 2 nucleoli (stage F2) (Faure et al. 2002) and showed a diffuse PCNA1-sGFP pattern. This observation is consistent with the detection of the G2/M marker CYCB1;2-YFP only 1 h after fertilization in the endosperm nucleus (Maruyama et al. 2015). Events of DNA replication in the egg cell upon sperm cell discharge and onward could not be obtained due to a limited expression of PCNA1-GFP (Fig. 3D, K, L, and M). A uniform to speckled pattern in the elongated zygote became apparent only once the endosperm reached the 4-to-8 nucleate stage (Fig. 3M to O; Supplemental Fig. S8). EdU staining experiments also indicated that replicated DNA was only detectable in zygotes accompanied by a 4 or 8-nucleate endosperm (Liu et al. 2021).

## Discussion

In this study, we confirm recent observations obtained by EdU staining (Liu et al. 2021) that mature male gametes in Arabidopsis are arrested in the G1 phase and further show that this block persists until their delivery into the ovule. Similarly, both female gametes are arrested in a pre-replicated state: the central cell in G1 phase and the egg cell in the G0 phase. The cell-cycle block is released upon fertilization for both male and female gametes. However, this release occurs asynchronously between the female gametes, as DNA replication occurs prior to karyogamy in the central cell but not in the egg cell, suggesting distinct underlying molecular mechanisms. This is further supported by the observation of autonomous proliferation in the central cell but not in the egg cell in the absence of fertilization in Polycomb group and *cyclin-dependent kinase A1 (cdka;1)* mutants (Guitton et al. 2004). Simonini et al. (2023) recently proposed a molecular mechanism that specifically unlocks central cell proliferation at fertilization. These results suggest that distinct, yet unknown, mechanisms operate in the egg cell to enable the initiation of DNA replication.

While a G1 arrest has been observed in Arabidopsis sperm cells (this study, Liu et al. 2021) and Torenia sperm cells, at anthesis (Liu et al. 2021), it is unclear if this is a universal trait among flowering plants. Interestingly, some flowering plants release bicellular pollen grains at anthesis and generate the 2 sperm cells exclusively during pollen tube growth. We have observed that Arabidopsis sperm cells remain in the G1 phase even during their transport in the pollen tube. This conclusion is likely to apply to bicellular angiosperms where there would not be sufficient time to generate the sperm cells and initiate their replication prior to fertilization. Future studies will determine whether a consistent G1-phase block is present in male gametophytes across all flowering plants.

## Materials and methods

### Plant materials and growth conditions

All Arabidopsis (*A. thaliana*) plants described in this study were in the Col-0 background (except when mentioned). The following reporter lines were described previously: pORC2-ORC2-GFP (Collinge et al. 2004) (Ler accession), pORC1a-ORC1a-GFP and pORC1b-ORC1b-GFP (Vergara et al. 2023), pLIG1-LIG1-GFP (Andreuzza et al. 2010), pHTR10-HTR10-Clover (Kawashima et al. 2014), pPCNA1-PCNA1-sGFP (Yokoyama et al. 2016), pH4-DB-CYCB1;1-Venus (R Jones et al. 2017), pCDT1a-CDT1a-GFP and pCDT1a-CDT1a-RFP (Desvoyes et al. 2019), pH1-1-H1-1-RFP (Rutowicz et al. 2019), and PlaCCI (Desvoyes et al. 2020). Reporter lines for all 4 *CYCB1* genes were provided by Arp Schnittger (Weimer et al. 2016). After a 4 d-stratification in the dark, seeds were germinated and grown on soil in a growth chamber under long days at 20 °C (16-h light/8-h night). For the purpose of collecting pollen for FACS, seeds were sown in 60 cm × 40 cm trays with 6 cm of soil (4:1 Gramoflor 2006:perlite), stratified for 4 d at 4 °C in the dark, and then moved to 21 °C/16 °C with 60% humidity, 16-h light/8-h night.

### Cloning and plant transformation

A PCNA2 genomic fragment comprising 412 bp upstream of the ATG until the last codon before the termination codon of the gene flanked with attL Gateway recombination sites was generated by gene synthesis (Genscript). The pPCNA1-PCNA1-TagRFP reporter construct was obtained after a LR Clonase II reaction using the pPCNA1-PCNA1 entry vector (Yokoyama et al. 2016) and the destination vector pGWB559 (Nakagawa et al. 2007). Similarly, the pPCNA2-PCNA2-TagRFP and pPCNA2-PCNA2-CFP constructs were generated using pGWB459 and pGWB543 destination vectors (Nakagawa et al. 2007), respectively.

The transgenic plants were generated by floral dipping (Clough and Bent 1998) and selected on MS solid medium (Duchefa) with the appropriate selective agent.

### Sample preparation and microscopy

Observations of the different reporter lines during male and female gametogenesis were obtained from freshly dissected anthers and carpels. Self-pollinated pistils expressing the PCNA1-sGFP marker (Yokoyama et al. 2016) or pistils pollinated with the PCNA1-sGFP and H1-1-RFP (Rutowicz et al. 2019) markers were dissected and mounted on ½ MS supplemented with 0.4% w/v Phytagel. All the steps ranging from pollination, growth of the pollen tubes in the pistil up to the double fertilization process, and consequent development of the fertilization products (zygote, endosperm) were reconstituted from a series of pistils dissected at distinct time after pollination (Ingouff et al. 2007). Semi–in vivo pollen tube growth was performed as described before (Palanivelu and Preuss 2006). Wild type pistils were pollinated with pollen grains expressing either the PCNA1-sGFP marker or a combination of ORC1b-GFP and CDT1a-RFP and cut at the end of the style. The pollinated pistils were incubated on a pollen tube germination medium at 25 °С for 4 h. Pollen tube growth was observed 4 to 6 h after pollination. Imaging of cell cycle reporters in roots was performed as previously described (Ingouff et al. 2017).

All the images were obtained using a laser scanning confocal microscope (Zeiss LSM880 Fast Airyscan) equipped with a 40X/1.1 water immersion objective and an Airyscan detector, in the super-resolution acquisition mode. Fluorescence was collected using the following settings for CFP (excitation 405 nm; emission band pass 420 to 480 nm), for GFP (excitation 488 nm; emission band pass 495 to 550 nm), for RFP (excitation 561 nm; emission band pass 570 to 620 nm), and for autofluorescence (excitation 488 nm; emission long pass 570 nm). Image contrast and brightness were adjusted with Adobe Photoshop, and processed images were assembled in Adobe Illustrator.

### Pollen collection and FACS

Pollen was harvested from open flowers using the vacuum suction method (Johnson-Brousseau and McCormick 2004), using 150 *μ*m, 60 *μ*m, and 10 *μ*m mesh filters and then flash frozen and kept in −80 °C. Sperm and vegetative nuclei were isolated by FACS following the previously described method (Borges et al. 2012). Collected pollen was hydrated by resuspension in an ice-cold Galbraith buffer (45 mM MgCl_2_, 30 mM Tri-Sodium Citrate, 20 mM MOPS pH 7.0, 0.1% Triton X-100) with added 72 mM ß-mercaptoethanol and 1× complete protease inhibitor cocktail (Roche) (Galbraith et al. 1983). Pollen suspension was then centrifuged for 1 min at 10,000, and 50 *μ*l of 100 *μ*m glass beads were added and vortexed for 4 min to disrupt pollen walls. The suspension was filtered through a 10 *μ*m nylon mesh to retrieve the sperm and the vegetative nuclei. Nuclei were then stained with SYBR Green (S9430, Sigma-Aldrich) or propidium iodide (P4170, Sigma-Aldrich).

A BD FACS AriaTM III Cell Sorter was used to sort the nuclei suspension. The sorter was operated according to standard configuration, utilizing a 70 *μ*m ceramic nozzle with a 1×Phosphate-Buffered Saline solution flowing at a constant 20 psi pressure. Sperm and vegetative nuclei were collected in 2 different tubes. For 2 identical replicates of the experiment with at least 1 wk difference between growth cycles, HTR10-Clover sperm nuclei were further separated in the FACS to 3 groups based on their propidium iodide fluorescence. The different experiments and sequenced samples are summarized in Supplemental Table S1.

### S-phase synchronization and release of MM2d cell suspension culture

MM2d cells from *A. thaliana* (Ler) were obtained from the lab of Crisanto Gutierrez and were previously described (Menges and Murray 2002, 2006). Cells were grown at room temperature in the dark shaking at 130 rpm and subcultured every week by adding 3 ml of the old culture into 50 ml fresh MSS media (1× MS, 3% sucrose 0.5 mg/l NAA, 0.05 mg/l kinetin). For cell cycle synchronization, 1-wk-old MM2d cells were diluted in new MSS media in a 1:5 ratio. After taking a sample for flow cytometry analysis, 4 *μ*g/ml of Aphidicolin (Sigma) was added to the diluted culture for the cell-cycle block. Cells were incubated for 24 h with shaking at 130 rpm at room temperature in the dark. After 24 h, a sample was taken for flow cytometry analysis and 40 ml of cells were harvested in liquid nitrogen. Cells were washed with MMS media to remove the Aphidicolin and were resuspended in media to resume the cell cycle synchronously. After 90 min, when cells are enriched for S phase, a sample was taken for flow cytometry analysis, and another 40 ml were harvested in liquid nitrogen. The experiment was done in 2 identical biological replicates, with growth cycles at least a week apart. For flow cytometry analysis, samples were chopped in Galbraith buffer (45 mM MgCl_2_, 30 mM Na-citrate, 20 mM MOPS pH 7.0, 0.1% Triton X-100) and filtered through a 40 *μ*m nylon mesh. Nuclei were stained with 4 *μ*g/ml DAPI and analyzed on the Penteon flow cytometer. FACS profiles were analyzed using flowCore (Hahne et al. 2009). To harvest the samples of G1 and 90 min following release into S phase, cells were filtered using a cell strainer, dried with filter sheets, and then flash frozen in liquid nitrogen. To break the cells, Precellys Zirconium oxide beads were added to the frozen cell pellets. Cells were disrupted by shaking 3 times at 5800 rpm for 20 s using the Precellys Evolution machine. Cells were frozen in liquid nitrogen between each disruption. DNA extraction, sonication of DNA, and construction of libraries for next-generation sequencing were done as for the sperm and vegetative nuclei.

### Isolation of DNA from nuclei and library preparation

Genomic DNA was extracted using QIAamp DNA Micro Kit (56304) following the provider “Small Volumes of Blood” protocol. DNA was diluted to 10 ng in 50 *μ*l of an elution buffer (10 mM Tris-HCl, pH 8.0) in microTube AFA Fiber tubes (520052). Samples were sonicated in Covaris E220 programed to 80 s/10 Duty/140 PIP/200 cycles. A total of 25 *μ*l of the sonicated DNA was used for library preparation using the NEBNext Ultra II DNA Library Prep Kit for Illumina (E7645S), with a final amplification of 12 PCR cycles. Libraries were sequenced on Illumina Novaseq or Nextseq machines, with at least 10 million paired-end reads per library.

### Processing of DNA-Seq libraries

All next-generation sequencing data used in this work were processed in the same way, including the previously published datasets. To avoid detecting effects attributed to technical differences in sequencing mode and coverage, sequence reads were down-sampled to 10^7^ paired-reads using seqtk v1.3 and trimmed to 50 bp for both R1 and R2 using Trim Galore v0.6.2 (–hardtrim5 50). Reads were then further trimmed using Trim Galore v0.6.2 (–paired –fastqc). Reads were then mapped to the TAIR10 genome using bowtie2 v2.3.5.1 with default parameters (Langmead and Salzberg 2012). Aligned reads were converted to indexed-sorted bam using samtools v1.10 (Danecek et al. 2021), and duplicate reads were filtered out using Picard v2.18.27 MarkDuplicates option. Coverage per 10 kb or 100 kb bins of the genome were calculated using DeepTools v3.3.1 (-of bedgraph) (Ramírez et al. 2016). The procedure described above was wrapped in a Nextflow pipeline (Di Tommaso et al. 2017), and was uploaded to GitHub. Genomic coverage per sample per bin size was then normalized to the same total signal and log2 transformed. Previously published data on genomic DNA from Col-0 roots (1001 Genomes Consortium 2016) were downloaded from SRA, accession number SRR1945757, and data for early, middle, and late S-phase cells labeled with EdU along with G1-phase cells (Concia et al. 2018) were downloaded from SRR3931891 to SRR3931900.

### Accession numbers

All sequencing data were deposited to SRA under accession number PRJNA914255. Accession numbers for the mentioned genes are as follows: CDT1a (At2g31270); CYCB1;1 (At4g37490); CYCB1;2 (At5g06150); CYCB1;3 (At3g11520); CYCB1;4 (At2g26760); Histone1-1 (At1g06760); HTR10 (At1g19890); LIG1 (At1g08130); ORC1a (At4g14700); ORC1b (At4g12620); ORC2 (At2g37560); PCNA1 (At1g07370); and PCNA2 (At2g29570).

## Supplementary Material

kiad512_Supplementary_Data

## Supplemental data

The following materials are available in the online version of this article.

**Supplemental Figure S1.** Synchronizing of cell suspension to G1 and release into S phase.

**Supplemental Figure S2.** DNA-Seq coverage along *A. thaliana* chromosomes.

**Supplemental Figure S3.** FACS sorting of sperm nuclei to 3 fractions.

**Supplemental Figure S4.** Dynamics of representative cell-cycle markers used in this study in root cells.

**Supplemental Figure S5.** Expression pattern of ORC2-GFP translational fusion in the mature pollen grain and embryo sac.

**Supplemental Figure S6.** Subnuclear pattern of PCNA1-sGFP and ORC1b-GFP; CDT1a-RFP in sperm cells in growing pollen tubes.

**Supplemental Figure S7.** Dynamics of cell-cycle reporters in the egg cell and the central cell in the mature embryo sac.

**Supplemental Figure S8.** Subnuclear pattern of PCNA1-sGFP in the developing zygote.

**Supplemental Table S1.** List of sequenced samples.

**Supplemental Table S2.** Expression of selected DNA replication genes in transcriptome of purified egg cell and central cell of Arabidopsis.
