# Supplementary material for: Cell cycle status of male and female gametes during Arabidopsis reproduction
Source: Plant Physiol. 2023 Sep 27;194(1):412–21. doi: 10.1093/plphys/kiad512 (PMC10756760; doi:10.1093/plphys/kiad512)
Supplement: kiad512_Supplementary_Data [file kiad512_supplementary_data.pdf]

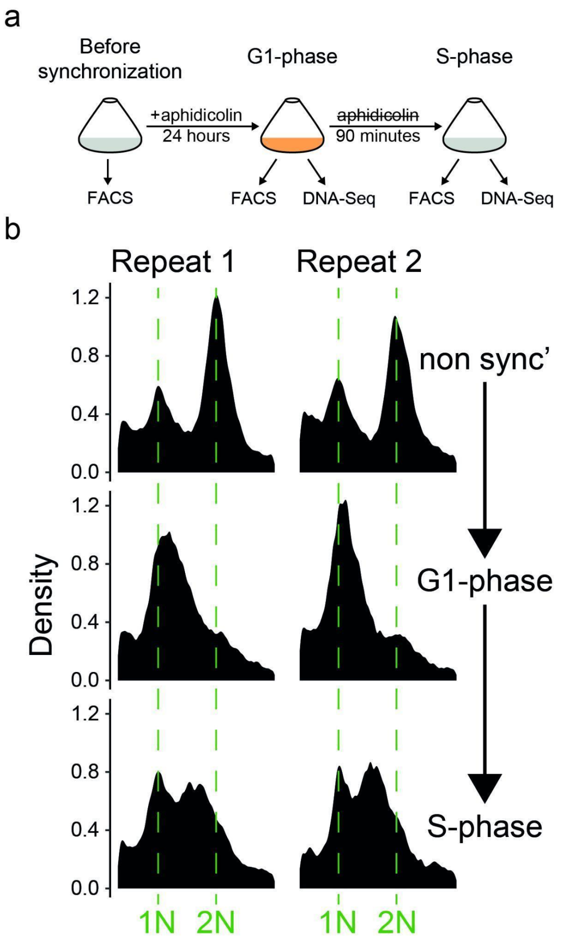

Supplementary Figure S1 | Synchronizing of cell suspension to G1 and release into S-phase. a, *A. thaliana* suspension cells were grown at room temperature in the dark, with shaking. These cells were synchronized to the G1 phase of the cell cycle by incubating them with aphidicolin for 24 hours. After this period, the aphidicolin was removed, allowing the cells to re-enter the cell cycle. Samples were collected at three different time points: before synchronization, after synchronization, and 90 minutes after the release from synchronization. At each time point, samples were collected for flow cytometry analysis, and DNA sequencing was performed on the samples collected at the G1- and S-phase time points. **b,** Fluorescence-activated cell sorting (FACS) profiles of DNA content measurements as quantified by DAPI staining shown at the three time points described in (**A**) for the two replicates of the experiment. Dashed lines represent haploid and diploid DNA content.

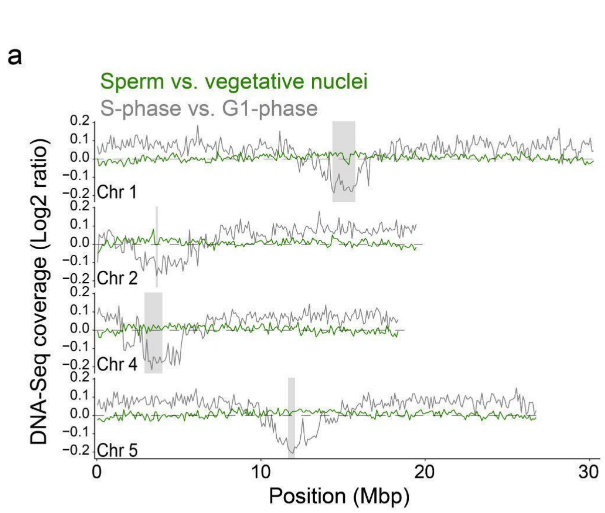

Supplementary Figure S2 | DNA-Seq coverage along *A. thaliana* chromosomes. a, DNA-Seq coverage as in Fig. 1b along chromosomes 1,2,4,5. **b,** The DNA-Seq coverage of sperm nuclei, normalized by DNA-Seq from root nuclei (1001 Genomes Consortium 2016), is shown for all five chromosomes of *A. thaliana* as in **(a)**. Gray shading indicates the centromere; dashed line marks the y-axis at 0.

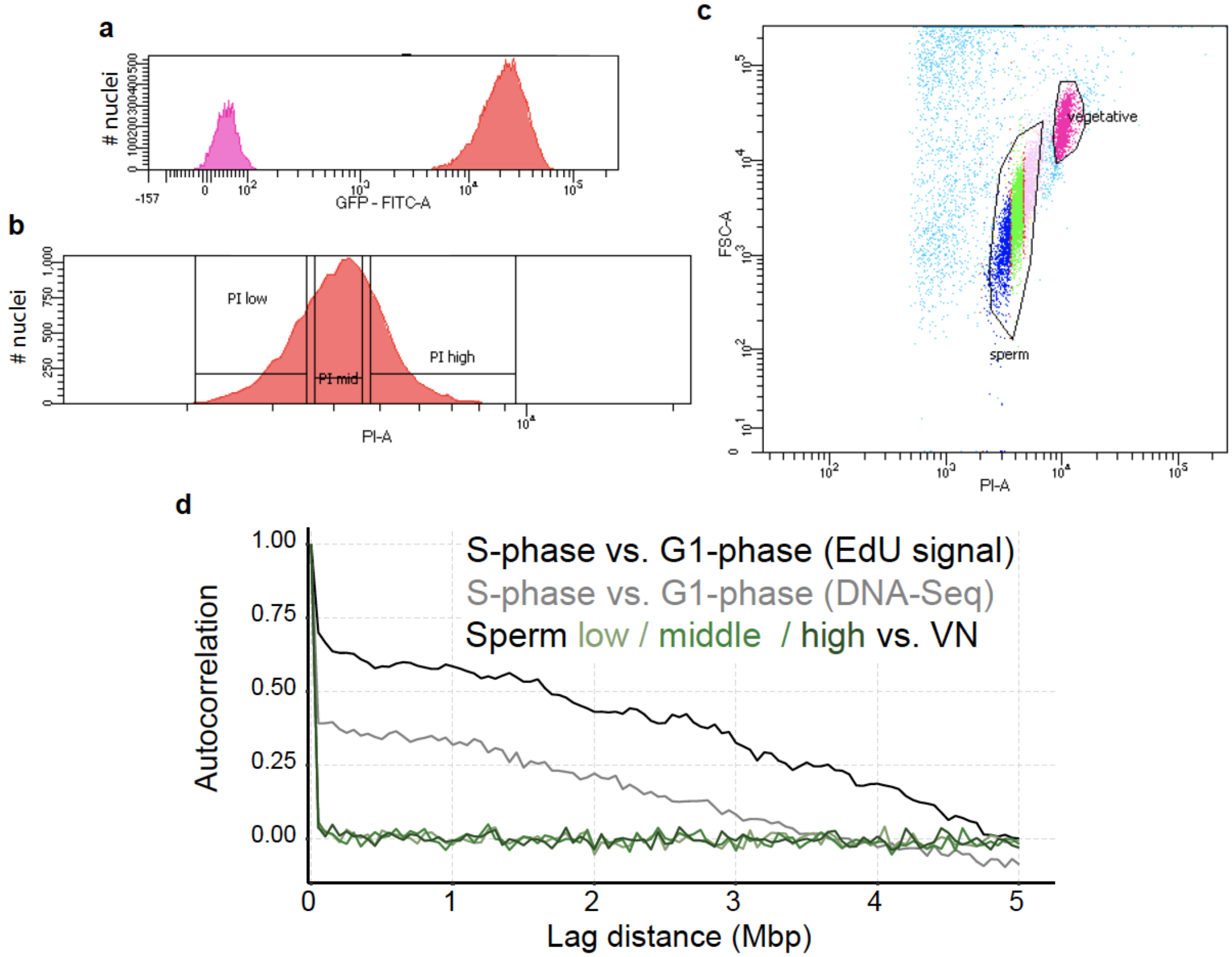

Supplementary Figure S3 | FACS sorting of sperm nuclei to three fractions. The sperm and vegetative nuclei in the HTR10-Clover sample were initially distinguished based on the Clover (GFP in axis) fluorescence (**a**). The sperm nuclei were then further divided into three fractions based on the DNA dye propidium iodide (PI), as shown in (**b**). The final sorting configuration, using PI, Clover, and forward scatter (FSC), is depicted in (**c**). **d,** The three fractions of sperm nuclei's autocorrelation (Pearson's correlation coefficient) are shown in different shades of green as shown in Fig. 1e. The S-phase vs. G1-phase autocorrelations are once more displayed for comparison, as in Fig. 1e. VN stands for vegetative nuclei.

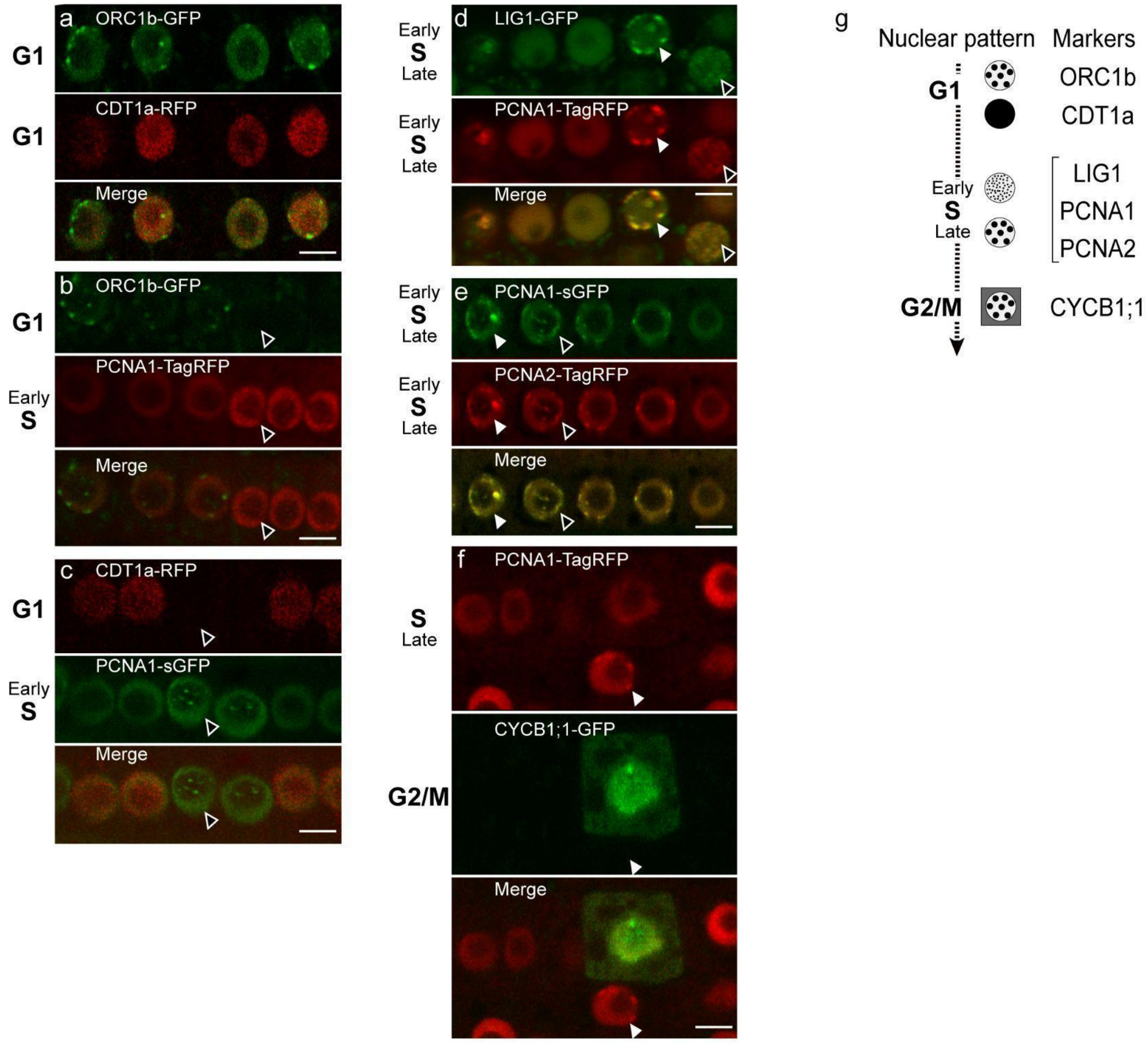

Supplementary Figure S4 | Dynamics of representative cell-cycle markers used in this study in root cells. Confocal images were obtained from cells of the root meristematic zone expressing a combination of two cell cycle phase markers. These markers all comprise the entire gene fused to a gene encoding a fluorescent protein. **a,** Dynamics of two G1 phase markers ORC1b-GFP and CDT1a-RFP. **b-c,** Dynamics of the S-phase marker PCNA1-TagRFP in combination with a G1 marker ORC1b-GFP **(b)** or CDT1a-RFP **(c)**. **d-e,** Dynamics of combined S-phase markers LIG1-GFP and PCNA1-TagRFP **(d)** or PCNA1-sGFP and PCNA2-TagRFP **(e)**. Dynamics of the S-phase marker PCNA1-TagRFP with the G2/M reporter CYCB1;1-GFP **(f)**. Empty and filled arrowheads indicate S-phase nuclei with dotted and speckled foci, respectively. They correspond to early and late S-phase nuclei, respectively. n>15 for each combination of markers. Scale bars, 10 µm. **g,** Diagram summarizing the typical nuclear patterns of the representative cell cycle phase markers used in this study. Circles filled with dots represent a focalized nuclear pattern. For the S-phase markers, circles filled with small dots or large dots indicate a dotted or speckled nuclear pattern, respectively. Filled black circles indicate a homogeneous nuclear pattern. A grey square symbolizes cytoplasmic accumulation.

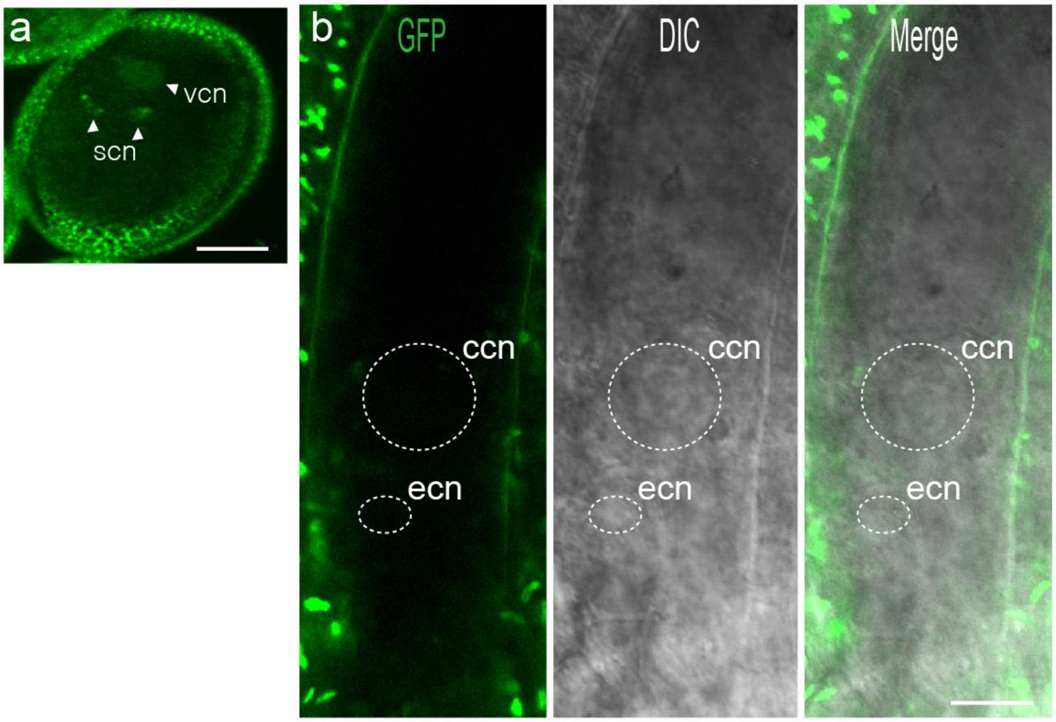

Supplementary Figure S5 | Expression pattern of ORC2-GFP translational fusion in the mature pollen grain and embryo sac. Confocal images were obtained from mature pollen grains **(a)** and embryo sacs **(b)** of plants expressing pORC2-ORC2-GFP (Collinge et al. 2004). **a,** A punctate fluorescent signal is detected in the sperm nuclei (n=35). **b,** No fluorescence is detected in the mature embryo sac (left panel, n=25). ccn, central cell nucleus; ecn, egg cell nucleus; scn, sperm cell nucleus; vcn, vegetative cell nucleus. Scale bars 10 µm **(a)** and 15 µm **(b)**.

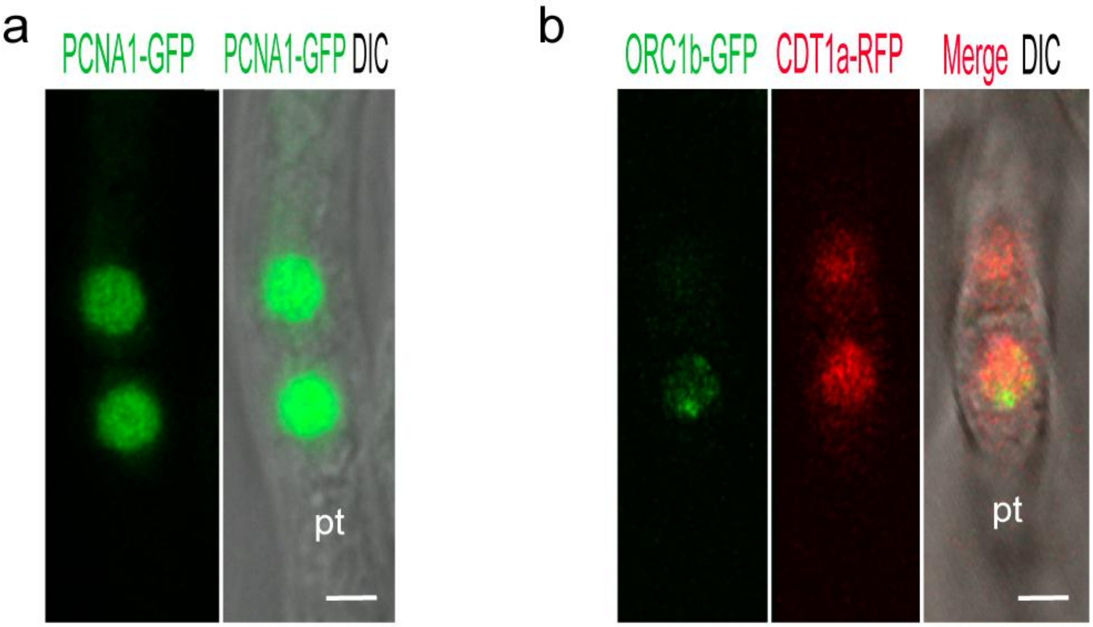

Supplementary Figure S6 | Subnuclear pattern of PCNA1-sGFP and ORC1b-GFP; CDT1a-RFP in sperm cells in growing pollen tubes. Wild type pistils were pollinated with pollen grains expressing the pPCNA1-PCNA1-sGFP reporter or a combination of ORC1b-GFP and CDT1-RFP markers and cut at the end of the style. Confocal images were taken from growing pollen tubes (pt) emerging from the style for 4 h after pollination (n=45). A uniform fluorescent signal is detected in the sperm nuclei in the growing pollen tube visualized by differential interference contrast (DIC). Scale bar, 10 μm.

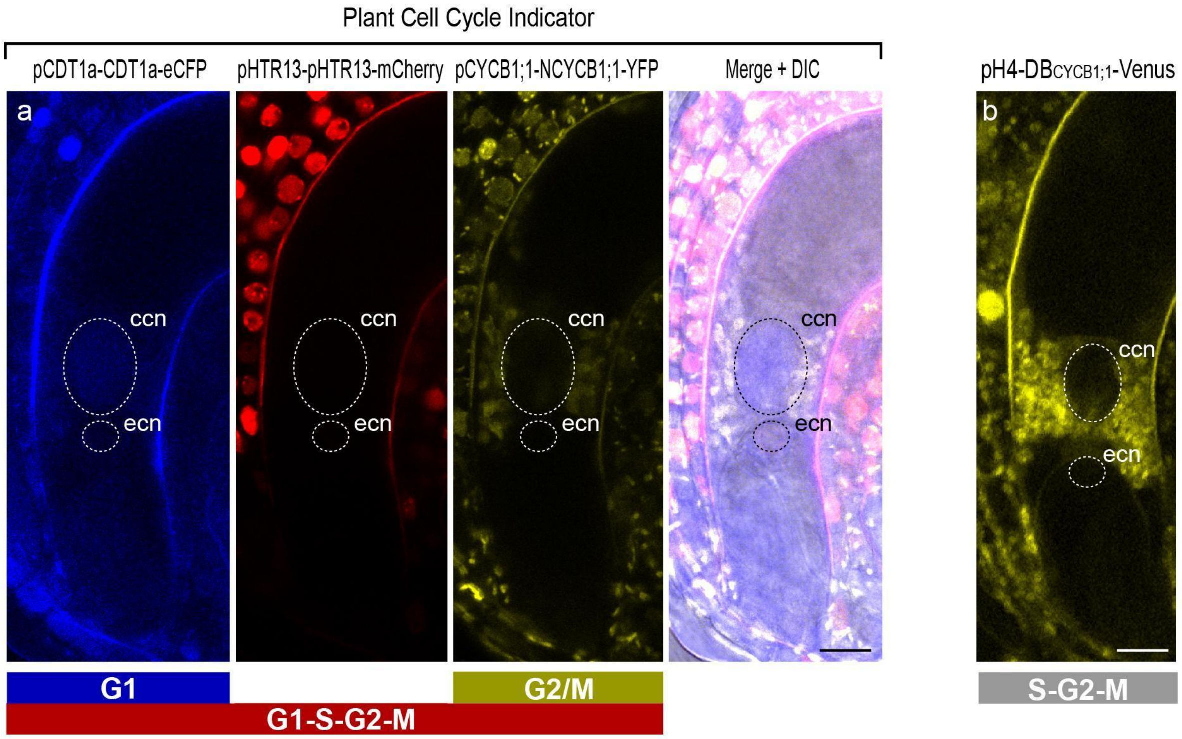

Supplementary Figure S7 | Dynamics of cell-cycle reporters in the egg cell and the central cell in the mature embryo sac. Confocal images obtained from mature embryo sacs from transgenic plants expressing either the Plant Cell Cycle (PlaCCI) Indicator sensor (Desvoyes et al. 2020) **(a)** or the S-G2-M phase fluorescent reporter (pH4-DB$_{CYCB1;1}$-Venus) (R Jones et al. 2017) **(b)**. **a,** Only a weak fluorescence of the G1 phase marker CDT1a-eCFP was detected in the central cell nucleus of the embryo sac expressing the PlaCCI sensor (n=15). None of the cell cycle phase markers were detected in the egg cell nucleus. **b,** No fluorescence was detected in the egg cell and central cell (n=25). The signals around the central cell nucleus correspond to the autofluorescence in panels a and b. ccn, central cell nucleus; ecn, egg cell nucleus. Scale bars, 15 μm.

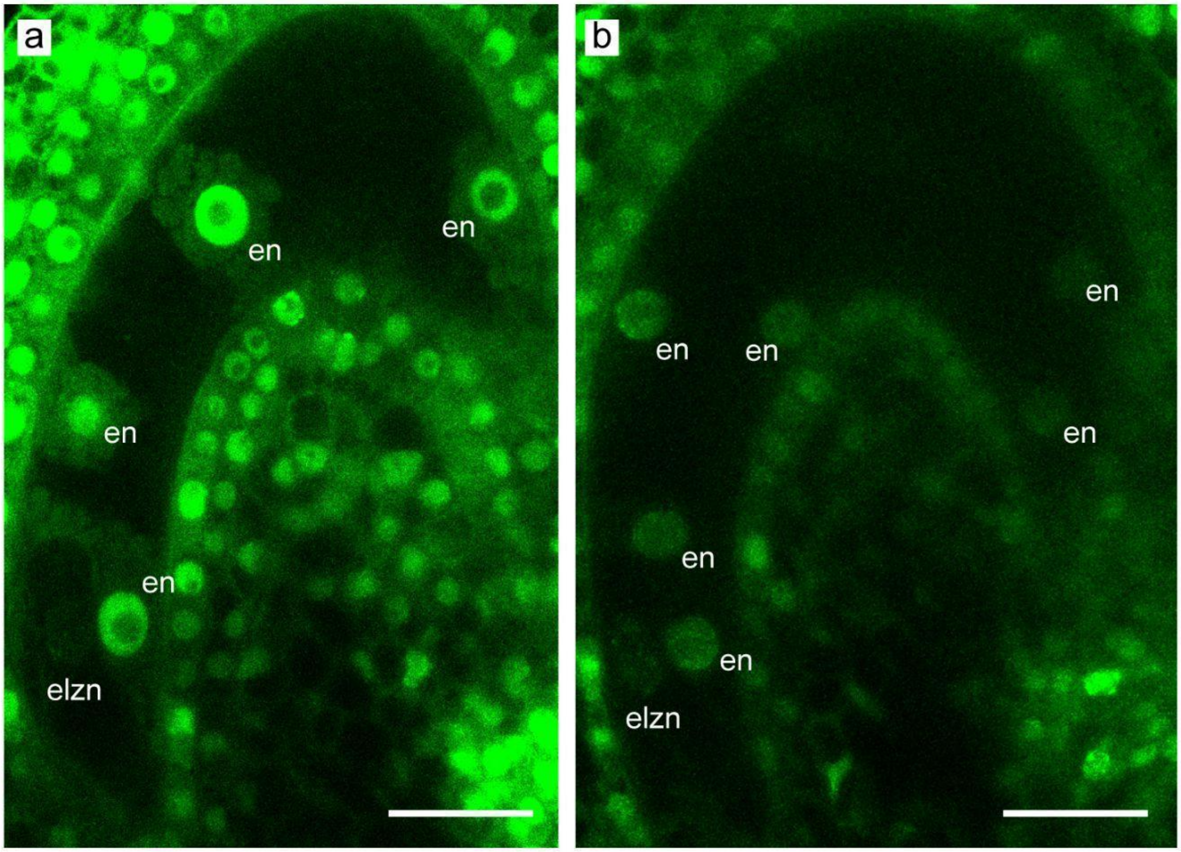

Supplementary Figure S8 | Subnuclear pattern of PCNA1-sGFP in the developing zygote. This supplementary figure is provided in support of Fig. 3. Fig. 3N and Fig. 3O are magnified views of the zygote in Fig. S8A and Fig. S8B, respectively. Confocal images have been generated from transgenic plants expressing pPCNA1-PCNA1-sGFP. At the 4-nuclear **(a, n=5)** and 8-nuclear endosperm stages **(b, n=5)**, the zygote becomes elongated and speckled foci of PCNA1-GFP are detected in the nucleus. elzn, elongated zygote nucleus; en, endosperm nucleus. Scale bars, 20 μm.

Supplementary Table S1 | List of sequenced samples.

SRA accession	Repeat	Genotype	Experiment setup	Sample
SAMN32329643	1	MM2d cell suspension	Aphidicolin synchronization	G1-phase
SAMN32329644	1	MM2d cell suspension	Aphidicolin synchronization	S-phase
SAMN32329645	2	MM2d cell suspension	Aphidicolin synchronization	G1-phase
SAMN32329646	2	MM2d cell suspension	Aphidicolin synchronization	S-phase
SAMN32329647	1	Col-0	Sort sperm/VN by SYBR-green	sperm
SAMN32329648	2	Col-0	Sort sperm/VN by SYBR-green	sperm
SAMN32329649	2	Col-0	Sort sperm/VN by SYBR-green	VN
SAMN32329650	3	Col-0	Sort sperm/VN by SYBR-green	sperm
SAMN32329651	3	Col-0	Sort sperm/VN by SYBR-green	VN
SAMN32329652	4	Col-0	Sort sperm/VN by SYBR-green	VN
SAMN32329653	4	Col-0	Sort sperm/VN by SYBR-green	sperm
SAMN32329654	1	Col-0	Sort sperm/VN by PI	sperm
SAMN32329655	1	Col-0	Sort sperm/VN by PI	VN
SAMN32329656	1	HTR10-Clover	Sort sperm by Clover (no DNA dye)	sperm
SAMN32329657	2	HTR10-Clover	Sort sperm/VN by Clover + PI	sperm
SAMN32329658	2	HTR10-Clover	Sort sperm/VN by Clover + PI	VN
SAMN32329659	3	HTR10-Clover	Sort sperm/VN by Clover + PI	sperm
SAMN32329660	3	HTR10-Clover	Sort sperm/VN by Clover + PI	VN
SAMN32329661	1	HTR10-Clover	Sort VN and sperm to 3 fractions by Clover + PI	VN
SAMN32329662	1	HTR10-Clover	Sort VN and sperm to 3 fractions by Clover + PI	Low sperm
SAMN32329663	1	HTR10-Clover	Sort VN and sperm to 3 fractions by Clover + PI	Middle sperm
SAMN32329664	1	HTR10-Clover	Sort VN and sperm to 3 fractions by Clover + PI	High sperm
SAMN32329665	2	HTR10-Clover	Sort VN and sperm to 3 fractions by Clover + PI	VN
SAMN32329666	2	HTR10-Clover	Sort VN and sperm to 3 fractions by Clover + PI	Low sperm
SAMN32329667	2	HTR10-Clover	Sort VN and sperm to 3 fractions by Clover + PI	Middle sperm
SAMN32329668	2	HTR10-Clover	Sort VN and sperm to 3 fractions by Clover + PI	High sperm

Supplementary Table S2 | Expression of selected DNA replication genes in transcriptome of purified egg cell and central cell of Arabidopsis.

		Egg cell				Central cell	
Gene Description	Gene ID	EC	EC1	EC2	EC3	CC1	CC2
		Zhao et al. 2019*	Susaki et al. 2021**			Susaki et al. 2021**	
Prereplication complex assembly							
ORC1A	At4g14700	2.2	0.1	2.2	0.0	1.8	15.8
ORC1B	At4g12620	0.0	5.5	22.4	5.1	14.4	16.8
ORC2	At2g37560	29.0	4.3	74.3	15.2	74.5	73.5
ORC3	At5g16690	3.1	3.4	0.0	0.0	19.0	30.3
ORC4	At2g01120	26.4	56.1	30.6	69.4	116.3	90.7
ORC5	At4g29910	27.1	1.6	0.0	38.5	71.3	36.8
ORC6	At1g26840	0.7	n.d.	n.d.	n.d.	n.d.	n.d.
NOC3	At1g79150	40.4	121.8	134.0	90.6	93.8	63.9
Cdc6a	At2g29680	0.00	0.0	0.0	0.0	48.8	96.0
Cdc6b	At1g07270	1.49	0.0	0.0	0.0	34.0	15.5
Cdt1a	At2g31270	8.10	49.1	43.1	0.0	149.0	115.7
Cdt1b	At3g54710	49.5	30.1	7.8	1.5	9.7	44.6
Cdc45	At3g25100	1.8	20.8	3.8	10.8	1.1	23.3
Mcm9	At2g14050	6.6	5.7	58.5	7.3	36.2	12.2
Replication fork assembly							
Mcm8	At3g09660	3.1	3.5	0.0	11.5	22.2	8.5
Mcm10	At2g20980	0.0	20.4	6.9	0.0	79.6	79.0
TOPBP	At1g77320	14.2	100.1	1.2	59.7	16.6	34.0
Psf1	At1g80190	0.8	0.0	0.0	0.0	108.9	30.4
Psf2	At3g12530	7.1	0.0	0.0	0.0	20.6	71.6
Psf3	At1g19080	27.7	69.0	94.4	108.0	144.1	37.8
	At3g55490	42.9	69.2	156.8	73.1	109.5	63.5
SLD5	At5g49010	7.0	5.5	0.4	4.5	4.0	3.1
DNA synthesis at replication forks							
POLA1	At5g67100	4.2	6.3	6.6	1.8	101.8	166.6
POLA2	At1g67630	0.0	11.2	0.0	0.0	108.5	23.1
POLA3	At1g67320	7.9	30.4	16.9	17.0	99.2	67.0
POLA4	At5g41880	1.3	0.0	0.0	0.0	99.7	40.4
POLD1	At5g63960	6.2	41.0	15.5	4.0	49.2	82.1
POLD2	At2g42120	57.5	10.6	10.9	8.2	7.3	88.0
POLD3	At1g78650	26.8	68.1	127.6	69.7	95.6	35.3
POLD4	At1g09815	37.1	29.7	0.0	4.9	0.0	57.4
POLE1	At1g08260	2.5	19.7	11.9	5.8	83.1	35.0
POL2B	At2g27120	2.5	25.1	9.0	17.5	59.4	44.4
POLE2	At5g22110	2.7	11.6	0.0	0.0	0.0	116.8
RPA1a	At2g06510	22.47	85.5	4.6	11.3	4.1	26.3
RPA1b	At5g08020	0.25	0.0	0.0	0.0	7.4	2.5
RPA1c	At5g45400	50.4	62.2	59.1	97.4	30.2	75.5
RPA1d	At5g61000	66.1	91.6	81.1	57.8	89.8	40.3
RPA1e	At4g19130	13.1	39.6	22.3	10.9	13.8	38.6
RPA2a	At2g24490	17.8	36.3	0.0	28.2	43.3	45.5
RPA2b	At3g02920	108.6	421.0	1101.8	253.9	79.3	149.8
RPA3a	At3g52630	0.0	0.0	0.0	0.0	71.0	36.9
RPA3b	At4g18590	59.9	22.0	4.7	0.0	71.3	3.7
PCNA1	At1g07370	13.4	24.1	2.5	0.0	78.5	119.4
PCNA2	At2g29570	15.9	43.6	0.0	81.2	93.4	63.1
LIG1	AT1G08130	23.9	17.8	53.6	20.2	193.8	188.5
Clamp loader							
RFC1	At5g22010	30.1	46.2	77.4	246.7	96.3	141.1
RFC2	At1g63160	16.6	31.8	0.0	0.0	24.8	22.0
RFC3	At5g27740	62.0	31.7	62.2	1.2	21.6	29.9
RFC4	At1g21690	39.8	3.8	26.8	15.1	30.8	80.2
RFC5	At1g77470	45.4	17.5	0.0	109.7	33.6	41.2

* Expression data from Zhao et al. doi.org/10.1016/j.devcel.2019.04.016
* Expression data from Susaki et al. doi.org/10.1371/journal.pbio.3001123

Supplemental references:

1001 Genomes Consortium. 2016. “1,135 Genomes Reveal the Global Pattern of Polymorphism in Arabidopsis Thaliana.” *Cell* 166 (2): 481–91.

Collinge, Margaret A., Charles Spillane, Claudia Köhler, Jacqueline Gheyselinck, and Ueli Grossniklaus. 2004. “Genetic Interaction of an Origin Recognition Complex Subunit and the Polycomb Group Gene MEDEA during Seed Development.” *The Plant Cell* 16 (4): 1035–46.

Desvoyes, Bénédicte, Ainhoa Arana-Echarri, María D. Barea, and Crisanto Gutierrez. 2020. “A Comprehensive Fluorescent Sensor for Spatiotemporal Cell Cycle Analysis in Arabidopsis.” *Nature Plants* 6 (11): 1330–34.

R Jones, Angharad, Manuel Forero-Vargas, Simon P. Withers, Richard S. Smith, Jan Traas, Walter Dewitte, and James A. H. Murray. 2017. “Cell-Size Dependent Progression of the Cell Cycle Creates Homeostasis and Flexibility of Plant Cell Size.” *Nature Communications* 8 (1): 15060.
